# Supplementary material for: Single versus repeated intravenous oncolytic reovirus infusions: Implications for immune modulation and rationalised scheduling of therapy in hepatocellular carcinoma
Source: Int J Cancer. 2026 Feb 16;158(12):3252–67. doi: 10.1002/ijc.70386 (PMC13106913; doi:10.1002/ijc.70386)
Supplement: Supplementary file 1 — APPENDIX S1: Supporting information. [file IJC-158-3252-s002.pdf]

Single versus repeated intravenous oncolytic reovirus infusions: Implications for immune modulation and rationalised scheduling of therapy in hepatocellular carcinoma

Karen J. Scott, Emma J. West, Rebecca J. Brownlie, Fay Ismail , Christy Ralph, Matt Coffey, Alan A. Melcher, Alison Taylor, Salvatore Papa, Adel Samson.

Contents:

Supplementary Table 1: Patient demographics for **(A)** repeated doses trial and **(B)** single dose trial.

Supplementary Table 2: Raw values (pg/ml) for cytokine analyses from **(A)** repeated doses and **(B)** single dose patients. Available as a separate Excel file.

Supplementary Table 3: Raw data values for immunophenotyping analyses from **(A)** repeated doses and **(B)** single dose patients. Available as a separate Excel file.

Supplementary Table 4: Raw data values for chemokine analyses from **(A)** repeated doses and **(B)** single dose patients. Available as a separate Excel file.

Supplementary Figure S1: Time course of activation of peripheral blood immune cells and chemokine/cytokine secretion in response to single or repeated doses of *i.v.* reovirus.

Supplementary Figure S2: Immune cell infiltration into livers and tumours in response to single or repeated doses of reovirus.

Supplementary Figure S3: Effect of a high fat diet on body weight and liver size of mice.

Supplementary Figure S4: Immunophenotyping of HCC patient PBMC in response to single or repeated doses of reovirus.

Supplementary Figure S5: Effect of a single dose of *i.v.* versus *i.t.* reovirus on PD-1, PD-L1 and CD69 expression by organ-resident T cells.

Supplementary Figure S6: Viability of HCC tumour and HCC cell lines in response to single or repeated doses of reovirus.

Supplementary Table 1: Patient demographics for **(A)** repeated doses trial and **(B)** single dose trial.

A

REPEATED DOSE TRIAL		
Age	Sex	Previous Therapy
74	M	Nil
62	M	Nil
73	M	Capecitabine + radiotherapy
66	M	Nil
62	M	Nil
50	F	Oxaliplatin
58	M	Nil
65	F	Nil
70	M	Nil
63	M	Nil

B

SINGLE DOSE TRIAL			
Age	Sex	Previous Therapy	Steroid Dose
59	M	Surgery + TMZ Chemoradiotherapy	8 mg
72	M	Oxaliplatin + radiotherapy	2 mg
45	F	Surgery + radiotherapy<br>PCV<br>Surgery	None
48	F	Surgery + radiotherapy<br>PCV<br>TMZ	None
64	M	Nil	2 mg
65	M	Surgery + TMZ Chemoradiotherapy	2 mg
65	M	Surgery + TMZ Chemoradiotherapy<br>CCNU<br>Bevacizamub	None
66	F	Surgery + TMZ Chemoradiotherapy	6 mg
74	F	Surgery	8 mg

TMZ: temozolomide; PCV: procarbazine; CCNU: lomustine

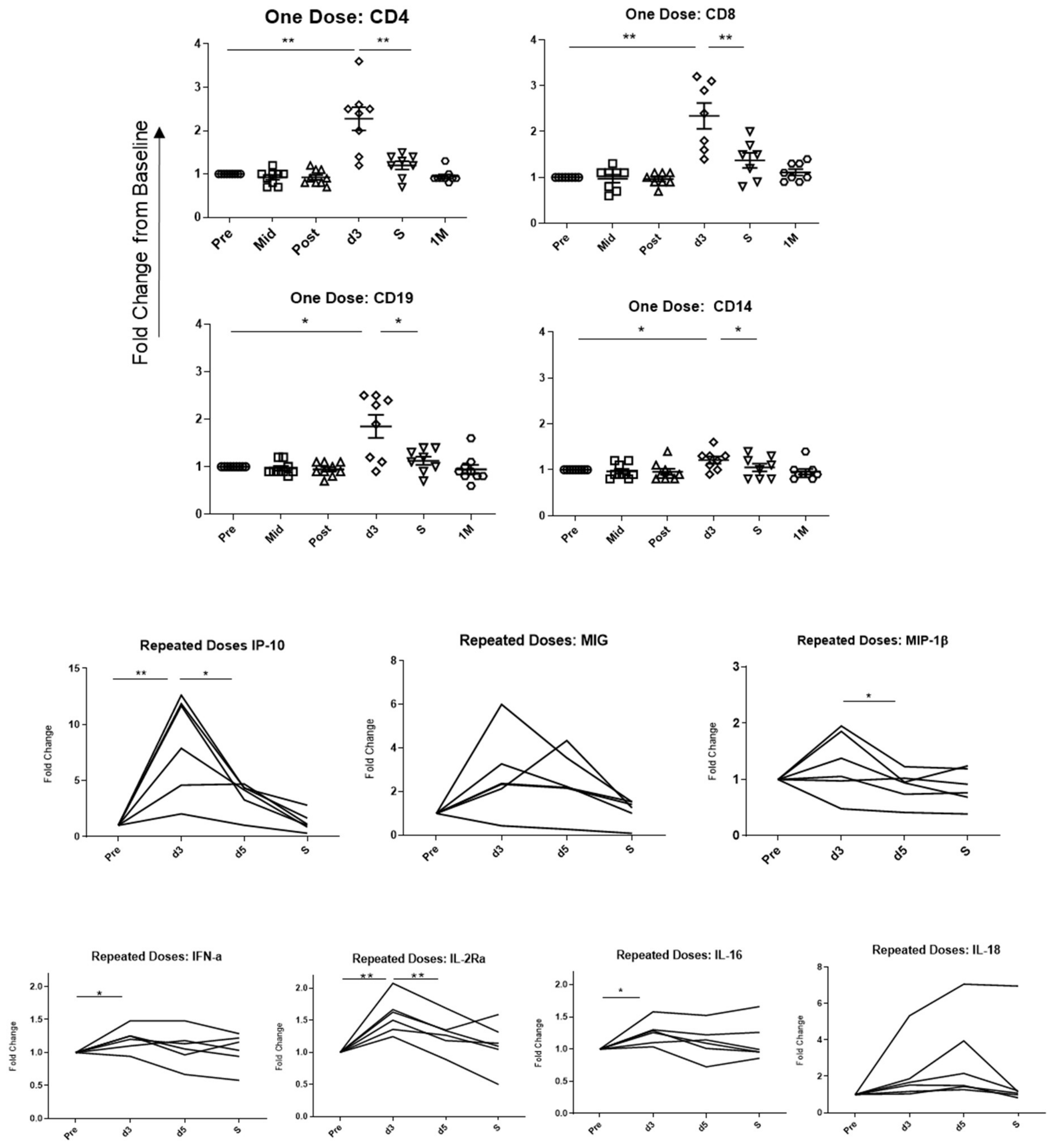

<u>Supplementary Figure S1: Time course of activation of peripheral blood immune cells and chemokine/cytokine secretion in response to single or repeated doses of *i.v.* reovirus.</u>

Whole blood immunophenotyping for cell-surface CD69 and multi-plex solute analysis on plasma was performed throughout study periods (including mid-infusion, 1hr post-infusion and day 5 samples). Data is presented as fold change from baseline (pre-infusion) sample; *P<0.05, **P<0.01. Repeated dose study: n=6; single dose study: n=9.

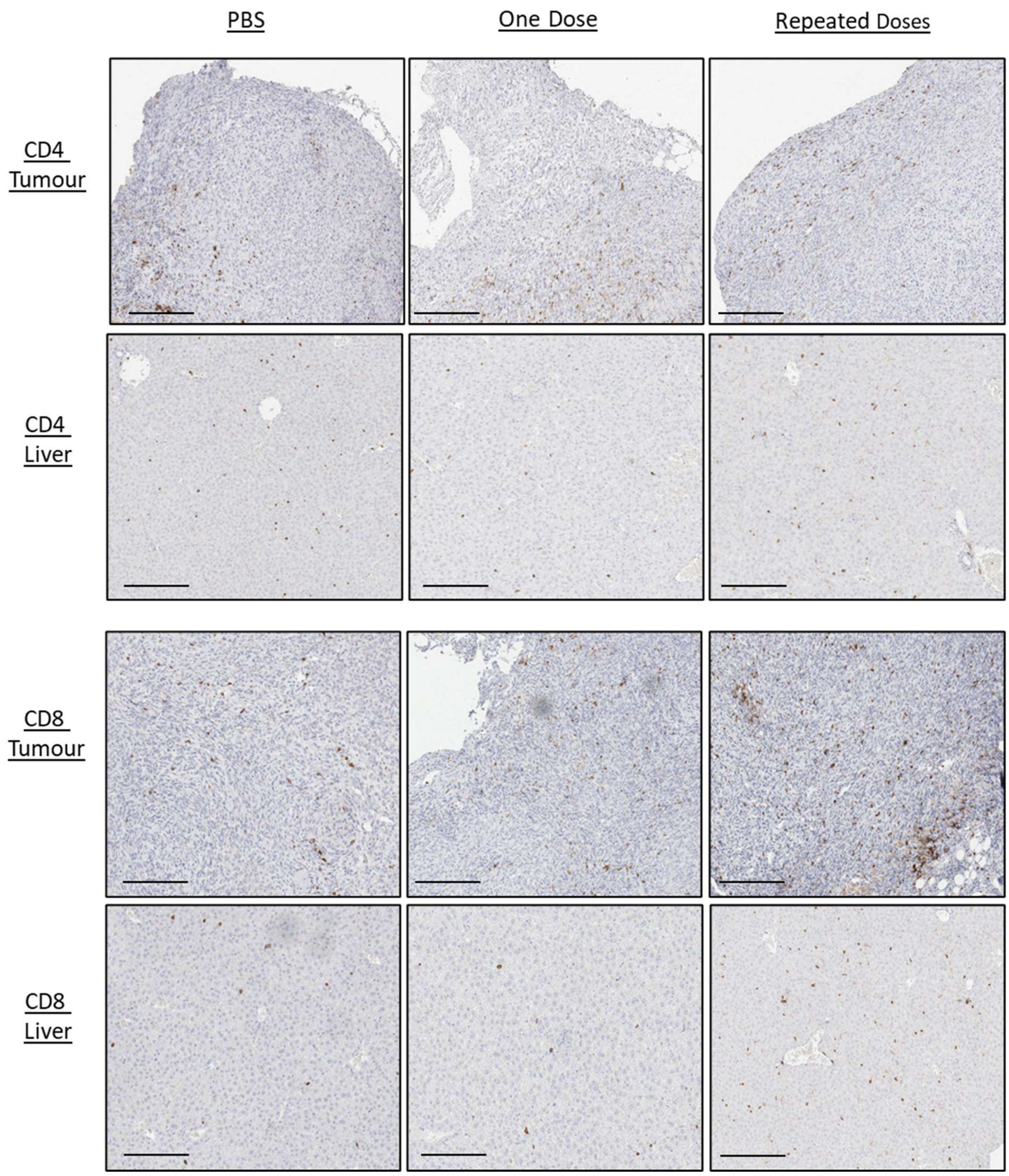

Supplementary Figure S2: Immune cell infiltration into livers and tumours in response to single or repeated doses of reovirus.

1MEA tumour-bearing mice were treated *i.v.* with PBS, a single or repeated doses of reovirus. Livers and tumours were harvested 72 hours after the final virus dose. IHC was performed on FFPE tissue for CD4+ and CD8+ T cells. Representative images are shown; positive staining is DAB (brown) and scale bars represent 100 µm.

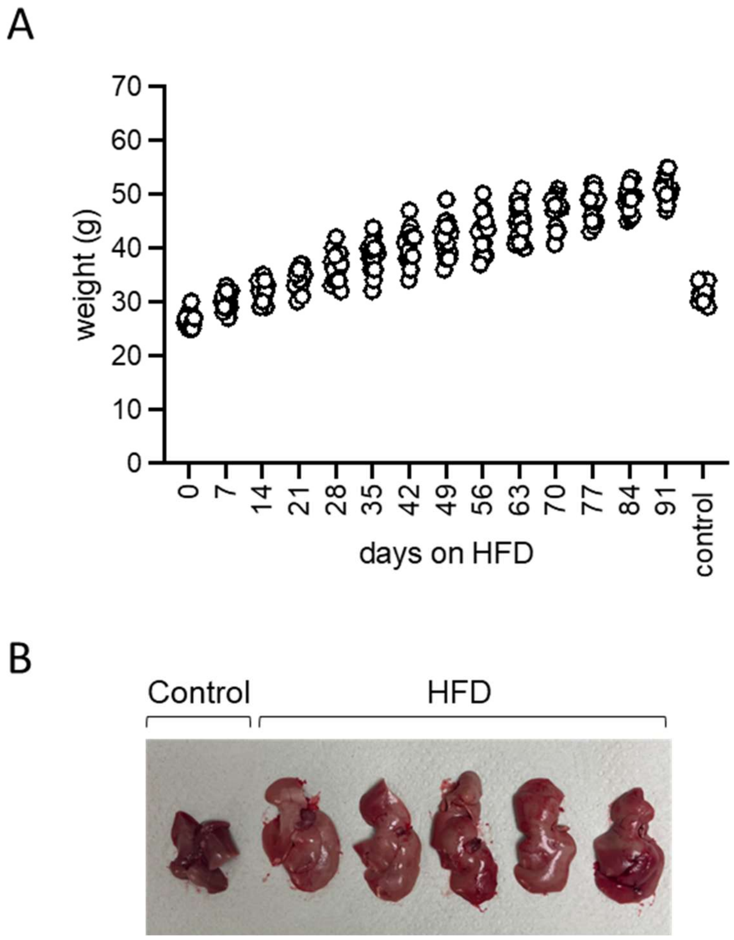

<u>Supplementary Figure S3: Effect of a high fat diet on body weight and liver size of mice.</u>

C57BL/6 mice were maintained on a HFD or kept on a normal diet as controls for 13 weeks prior to reovirus treatment. **(A)** Mice weights were recorded weekly (n=7 per group). **(B)** Photographs of an example control liver compared to HFD livers.

A

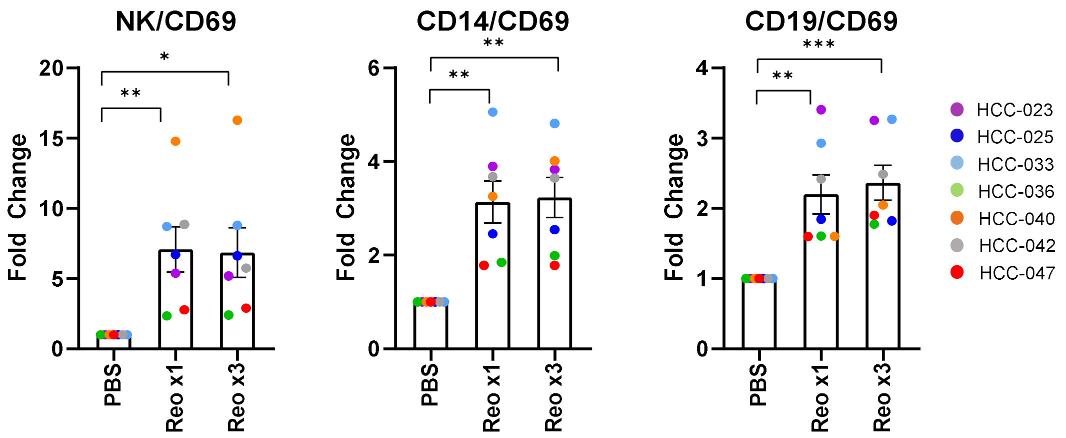

B

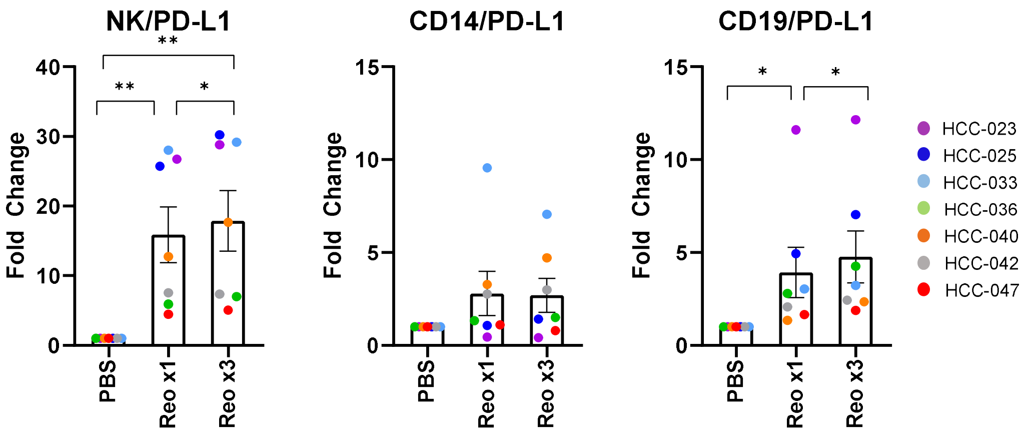

<u>**Supplementary Figure S4: Immunophenotyping of HCC patient PBMC in response to single or repeated doses of reovirus.**</u>

PBMCs were isolated from the blood of patients with HCC. Cells were treated with PBS, one (Reox1) or repeated (Reox3) doses of reovirus. After 24 hours of culture, immunophenotyping for cell-surface **(A)** CD69 and **(B)** PD-L1 was performed. Data is presented as fold change (± SEM) from PBS sample; *P<0.05, **P<0.01, ***P<0.001; n=7.

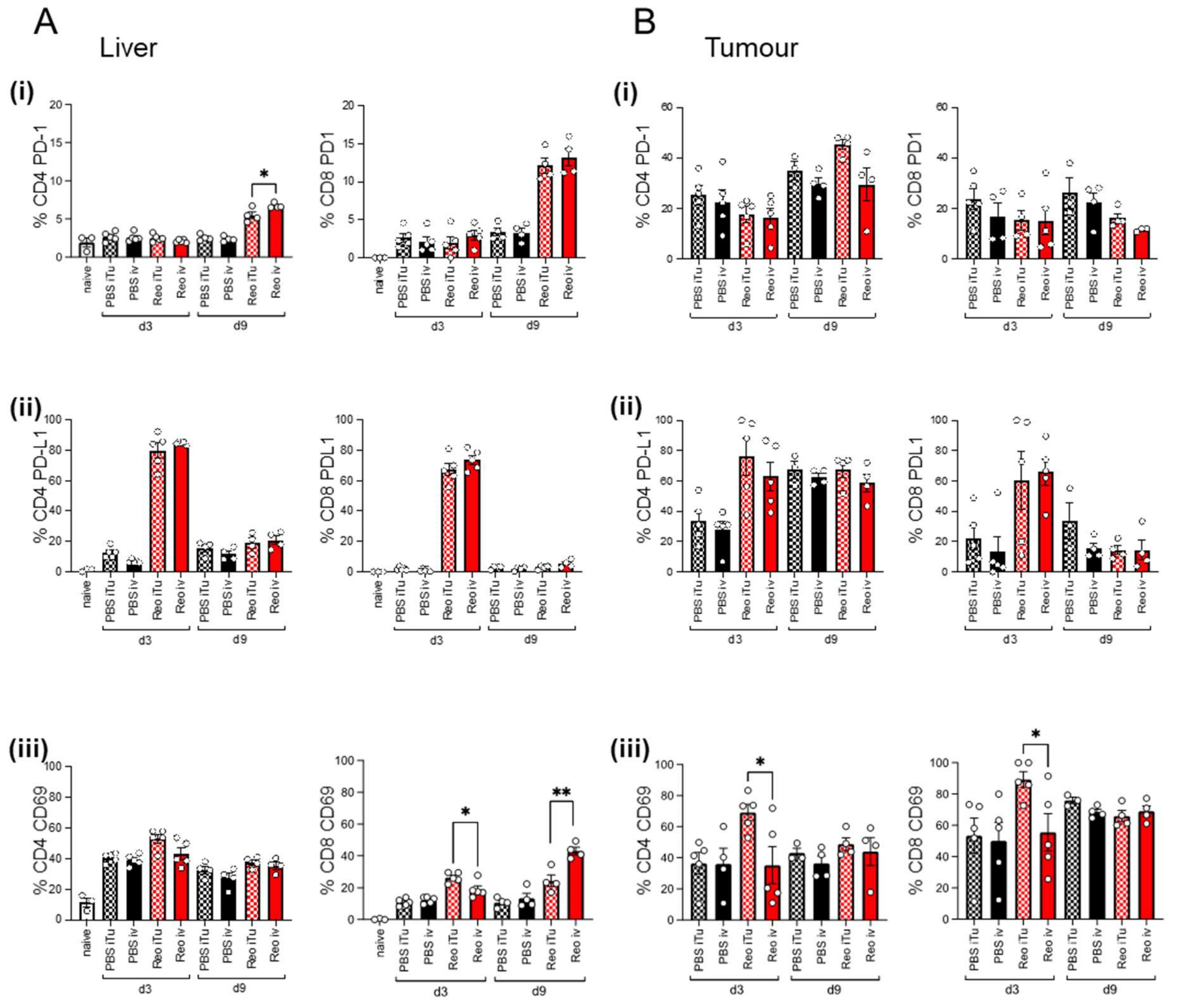

Supplementary Figure S5: Effect of a single dose of *i.v.* versus *i.t.* reovirus on PD-1, PD-L1 and CD69 expression by organ-resident T cells.

1MEA tumour-bearing mice were treated with a single dose of *i.v.* or *i.t.* reovirus or PBS (naïve represents untreated mice) prior to organ harvest at 3 or 9 days post-treatment. Single cell suspensions of liver (A) and tumour (B) were analysed by flow cytometry for (i) PD-1, (ii) PD-L1 and (iii) CD69 expression on CD4+ and CD8+ T cells. Data is presented as mean % positive ± SEM for n=5 mice per group for naïve (white bars), PBS (black bars) and reovirus (red bars) for *i.v.* reovirus (solid bars) or *i.t.* reovirus (hatched bars); *P<0.05, **P<0.01.

A

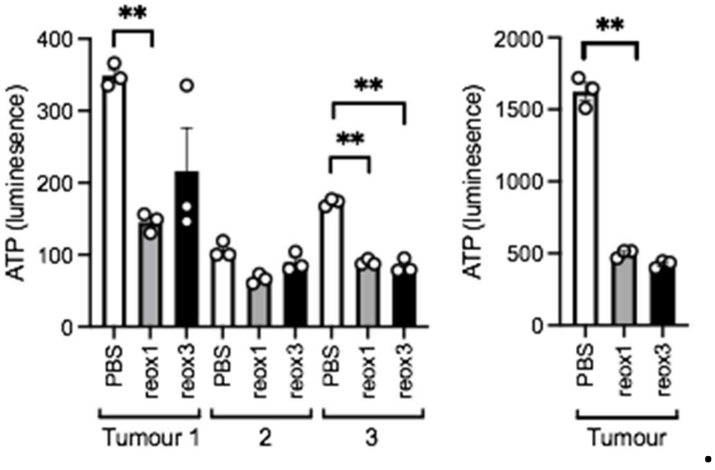

B

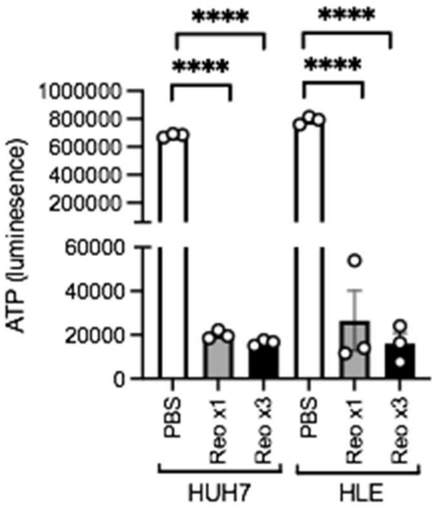

<u>Supplementary Figure S6: Viability of HCC tumour and HCC cell lines in response to single or repeated doses of reovirus.</u>

(A) Single-cell suspensions of HCC tumour and **(B)** HCC cell lines were treated with PBS (white bars), one (Reox1; grey bars) or repeated doses (Reox3; black bars) of reovirus. After 24 hours of culture, ATP levels within cultures were measured. Data is presented as luminescence ± SEM from n=3 HCC tumours from one individual patient and n=1 tumour from a different patient; **P<0.01, ****P<0.0001.
